# Enhancing Kidney Quality Assessment: Power Doppler During Normothermic Machine Perfusion

**DOI:** 10.1111/aor.14983

**Published:** 2025-03-12

**Authors:** Yitian Fang, Anton V. Nikolaev, Jeroen Essers, Gisela Ambagtsheer, Marian C. Clahsen‐van Groningen, Robert C. Minnee, Ron W. F. de Bruin, Gijs van Soest

**Affiliations:** ^1^ Division of HPB and Transplant Surgery, Department of Surgery, Transplant Institute Erasmus Medical Center Rotterdam the Netherlands; ^2^ Cardiovascular Institute, Thorax Center, Department of Cardiology Erasmus Medical Center Rotterdam the Netherlands; ^3^ Department of Molecular Genetics, Cancer Genomics Center Erasmus Medical Center Rotterdam the Netherlands; ^4^ Department of Radiotherapy Erasmus Medical Center Rotterdam the Netherlands; ^5^ Department of Vascular Surgery, Cardiovascular Institute Erasmus Medical Center Rotterdam the Netherlands; ^6^ Department of Pathology and Clinical Bioinformatics Erasmus Medical Center Rotterdam the Netherlands; ^7^ Department of Medicine 2 (Medical Faculty) RWTH Aachen University Aachen Germany; ^8^ Department of Precision and Microsystems Engineering, Faculty of Mechanical Engineering Delft University of Technology Delft the Netherlands; ^9^ Wellman Center for Photomedicine Massachusetts General Hospital Boston Massachusetts USA

**Keywords:** Doppler ultrasound, experimental animal model, kidney function, quality assessment

## Abstract

**Objectives:**

Marginal donor kidneys are increasingly used for transplantation to overcome organ shortage. This study aims to investigate the additional value of Power Doppler (PD) imaging in kidney quality assessment during normothermic machine perfusion (NMP).

**Methods:**

Porcine kidneys (*n* = 22) retrieved from a local slaughterhouse underwent 2 h of NMP. Based on creatinine clearance (CrCl) and oxygen consumption (VO_2_) at 120 min, kidneys were classified into Group 1 (*n* = 7, CrCl > 1 mL/min/100 g and VO_2_ > 2.6 mL/min/100 g) and Group 2 (*n* = 15, CrCl ≤ 1 mL/min/100 g and/or VO_2_ ≤ 2.6 mL/min/100 g). PD imaging was performed at 30, 60, and 120 min, and PD metrics, including vascularization index (VI), flow index (FI), and vascularization flow index (VFI) were calculated. Renal blood flow (RBF), CrCl, and VO_2_ were measured at the same time points during NMP. The metrics were compared utilizing correlation analysis.

**Results:**

FI and VFI moderately correlated with CrCl (*r* = 0.537, *p* < 0.0001; *r* = 0.536, *p* < 0.0001, respectively), while VI strongly correlated with VO_2_ (*r* = 0.839, *p* < 0.0001). At 120 min, PD metrics demonstrated the highest diagnostic accuracy for distinguishing between the two groups, with an area under the curve (AUC) of 0.943 for VI, 0.924 for FI, and 0.943 for VFI. Cutoff values of 17% for VI, 50 a.u. for FI, and 9 a.u. for VFI provided 100% specificity and 73% sensitivity in identifying kidneys in Group 2, with an overall diagnostic accuracy of 82%. Baseline kidney biopsies showed moderate acute tubular necrosis in both groups, with no significant differences.

**Conclusions:**

PD metrics strongly correlate with renal viability and effectively differentiate kidneys with higher and lower functionality during NMP. PD imaging can be a valuable alternative to RBF during NMP for kidney quality assessment.

## Graphical abstract

Power Doppler imaging was evaluated as a non‐invasive tool for assessing kidney quality during normothermic machine perfusion. Power Doppler metrics demonstrated high accuracy in identifying kidneys with low function and viability (Group 2), highlighting its potential to enhance transplant decision‐making when integrated with existing assessment methods.

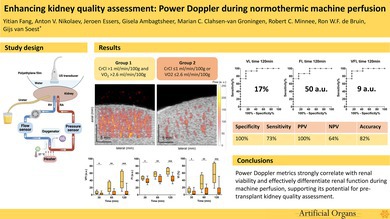

## 1 Introduction

Kidney transplantation is considered the optimal treatment for end‐stage renal disease [1]. Due to persistent organ shortages, kidneys from donation after circulatory death (DCD) or expanded criteria donors (ECD) are increasingly used for transplantation. Unfortunately, such kidneys are associated with a high risk of graft failure due to the suboptimal conditions of the donors, such as old age, uncertain medical history, long ischemia time, or pre‐donation acute kidney injury [2, 3]. To reduce the risk of graft failure, organ quality has to be carefully assessed before transplantation, mainly based on procurement biopsies, despite its invasive nature [4].

Normothermic machine perfusion (NMP) has emerged as an experimental preservation technique which aims to restore and assess kidney function by supplying the organ with perfusate enriched with nutrients and oxygen at 35°C–37°C to simulate the physiological environment of the human body [5, 6]. This innovative platform allows for the evaluation of renal functional capacities and injury through various parameters, including renal blood flow (RBF), intrarenal resistance (IRR), urine output, creatinine clearance (CrCl), oxygen consumption (VO_2_) and optical imaging during NMP [7, 8].

Markgraf et al. utilized hyperspectral optical imaging (HSI) to measure renal cortex oxygen saturation and water content as indicators of tissue damage [9, 10]. Schuller et al. used magnetic resonance imaging (MRI) to assess renal blood distribution during NMP [11, 12]. Our previous research demonstrated the value of laser speckle contrast imaging (LSCI) and photoacoustic imaging (PAI) to visualize renal cortical microcirculation and assess kidney quality [13, 14].

Alternatively, renal perfusion can be assessed using Ultrasound (US) Power Doppler (PD). Based on the Doppler effect, PD measures the amplitude of the Doppler signal and visualizes blood flow patterns [15, 16]. PD imaging has been widely used in various clinical scenarios to assess blood flow, vascular structures, and hemodynamic abnormalities [17, 18, 19, 20]. In kidney transplant patients, PD is mainly used to detect vascular complications, early acute rejection, and renal dysfunction [21, 22, 23].

Despite its use in post‐transplant practice, no study has reported PD imaging in assessing organ quality during pre‐transplant NMP. Recognizing this gap, our study aims to investigate the additional value of PD imaging in kidney quality assessment during NMP and its correlation with function and viability markers.

## 2 Materials and Methods

### 2.1 Study Design

Twenty‐two kidneys from 6‐month‐old female Landrace pigs were harvested from a local slaughterhouse. The animals were exsanguinated before slaughter, which simulates DCD conditions. Next, kidneys were transported to the laboratory and underwent 2 h of NMP for quality assessment.

During NMP, traditional functional parameters, including CrCl and VO_2_, and PD data were acquired at 30, 60, and 120 min of NMP. Kidneys were classified into two groups based on the CrCl and VO_2_ values at 120 min. Specifically, Group 1 included kidneys with CrCl > 1 mL/min/100 g and VO_2_ > 2.6 mL/min/100 g, while Group 2 included kidneys with CrCl ≤ 1 mL/min/100 g and/or VO_2_ ≤ 2.6 mL/min/100 g. These thresholds were established based on previous studies utilizing similar NMP protocols and perfusate compositions. Although the kidneys were not transplanted, those with relatively low CrCl and VO_2_ during NMP may be associated with poor post‐transplant function [24, 25].

Since the kidneys were obtained from pigs intended for meat consumption, no approval from the animal ethics committee was required.

### 2.2 Kidney Preparation

Back‐table procedures have been described elsewhere [13]. Briefly, two liters of blood were collected in a heparinized bucket and filtered by leukocyte filter (BioR 02 plus, Fresenius Kabi, the Netherlands) during exsanguination. The kidneys were then explanted by a designated butcher following a standardized procedure. Once the kidneys were explanted, back‐table preparation was performed by two of the authors (Y.F. and G.A). The renal artery was cannulated and flushed with 500 mL 4°C Ringer's lactate (Baxter BV, Utrecht, the Netherlands). During transportation to the laboratory, kidneys were preserved using hypothermic machine perfusion (LifePort, Organ Recovery Systems, USA) until NMP. Warm ischemia time (WIT) was calculated from the blood collection until cold flushing. Cold ischemia time (CIT) was defined as the hypothermic machine perfusion period.

Before NMP, the kidneys were flushed with 100 mL of 4°C Ringer's lactate to remove the cold preservation solution. Subsequently, the kidneys underwent 2 h of NMP with an autologous blood‐based solution (Table S1) at 37°C with a controlled pressure of 70 mmHg. Carbogen (95% O_2_ and 5% CO_2_) was supplied via the oxygenator at a flow rate of 0.5 L/min.

### 2.3 Experimental Setup

**FIGURE 1:**
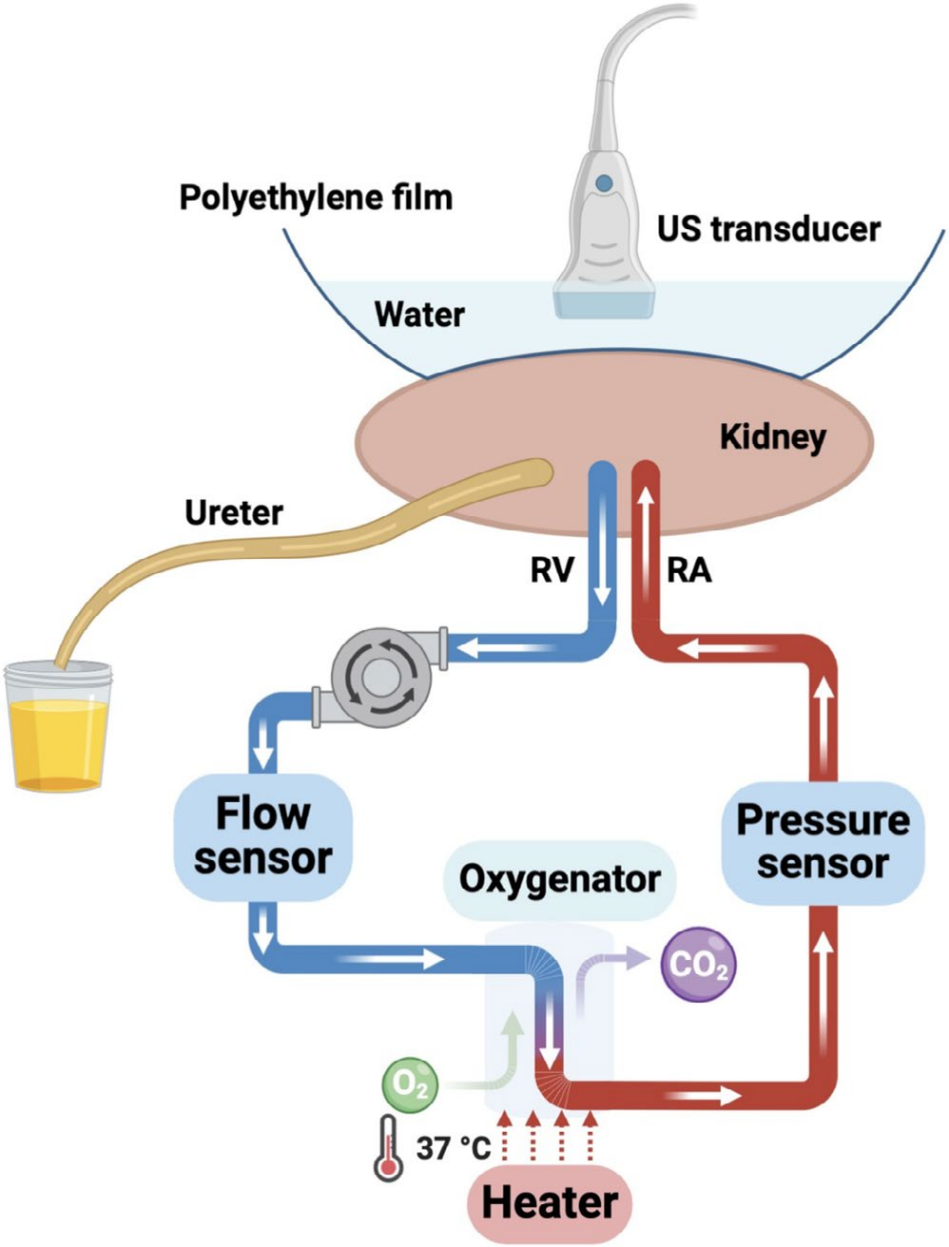
Schematic of the normothermic machine perfusion (NMP) and ultrasound (US) setup. RA, renal artery; RV, renal vein. [Color figure can be viewed at wileyonlinelibrary.com]

The schematic of the perfusion and measurement setup is depicted in Figure 1.

The NMP system consists of a centrifugal pump head (BPX‐80 Bio‐Pump Plus, Medtronic, Minneapolis, USA) equipped with a pump drive (BVP‐BP, Harvard Apparatus, Germany), an oxygenator with a heat exchanger (Hilite 1000, Medos, Germany), and a thermocirculator (E100, Lauda, Germany). A flow probe (73–4755, Harvard Apparatus, Germany) is connected in‐line with the kidney, and a pressure sensor (APT300, Harvard Apparatus, Germany) is directly connected to the 5 mm straight cannula (Organ Recovery Systems, USA). The set‐up is controlled by a commercially available electronic controller (PLUGSYS Servo Controller for Perfusion, Harvard Apparatus, Germany) which enables both flow and pressure‐directed perfusions.

The PD system, a Vevo3100 ultrasound (Fujifilm VisualSonics, Canada) system, is equipped with an MX201 US linear transducer array (VisualSonics, Canada) operating at a 15 MHz central frequency. During the PD acquisition, the transducer was attached to a linear translational stage, enabling 3D image acquisition. To provide the acoustic coupling, we covered the kidney with a polyethylene film filled with water as the coupling media.

### 2.4 Renal Function, Viability, and Injury Measurement

RBF was monitored by the flow sensor continuously throughout NMP. The perfusate samples from the renal artery and vein were collected at 30, 60, and 120 min of NMP for blood gas analysis (epoc Blood Analysis System, Siemens Healthineers, Canada). The urine output was recorded and collected at the same time points for further measurement.

CrCl, defined as the volume of perfusate plasma that is cleared of creatinine per unit time, is used as a renal function marker. VO_2_, defined as the amount of oxygen consumed per unit time, is used as a renal viability marker [26].

Baseline kidney biopsies were taken before NMP and fixed in 4% buffered paraformaldehyde. The tissue samples were then embedded in paraffin and cut into 5 μm sections. Using periodic acid‐Schiff (PAS) staining, acute tubular necrosis (ATN) was evaluated and scored on a semiquantitative scale of 0 to 3 (0‐no changes, 1‐mild, 2‐moderate, 3‐severe changes) by an experienced renal pathologist (M.C.v.G.) blinded to the study. The ATN score was based on the degree of brush border loss, tubular dilatation, epithelial vacuolation, thinning and sloughing, and luminal debris/casts.

### 2.5 Power Doppler Measurement

PD data were acquired from the lateral aspect of the renal cortex at 30, 60, and 120 min of NMP. To achieve this, the kidney was positioned laterally, and the US transducer was positioned directly above the kidney. The transducer was automatically translated over a range of 45 mm by 0.5 mm steps. The settings for the PD measurements are reported in Table S2, which were chosen manually to optimize the visualization of the renal cortical vasculature during NMP.

PD data were used to calculate vascularization index (VI), flow index (FI), and vascularization flow index (VFI). VI was defined as the ratio of colored pixels to all pixels within the region of interest (ROI), indicating the number of blood‐filled vessels. FI was defined as the mean PD signal intensity from all colored pixels, indicating the blood volume at the time of measurement. VFI is the combination of vascularization and blood volume, mathematically multiplying VI by FI [27]. The equations are shown as below:

(1) $$\mathrm{VI}\;\left(\%\right)=\frac{\#\text{colored pixels}}{\#\text{total pixels}}\times 100\%$$

(2) $$\mathrm{FI}\left(a.u.\right)=\frac{\sum_{i=1}^{\#\text{colored pixels}}w\left(i\right)}{\#\text{colored pixels}}$$

(3) $$\mathrm{VFI}\left(a.u.\right)=\mathrm{VI}\times \mathrm{FI}$$

where $w\left(i\right)$ is a value of the *i* ^*th*^ colored pixel which represents intensity of the PD signal. The pixel value represents power of the Doppler signal and normalized based of the settings and scaled between 0 and 255. The ROI is defined as the renal cortical layer at a depth of 5 mm from the kidney surface, as the majority of functional nephrons are located within this cortical layer. For further data analysis we calculated the mean value of each metric within the acquired volume.

### 2.6 Statistical Analysis

We reported continuous variables using median and interquartile range (IQR). Renal function, viability, and PD metrics at each time point were compared between groups using the Mann–Whitney U test. The correlations between PD metrics and functional parameters, as well as between RBF and functional parameters, were investigated using the Spearman's linear correlation test. Receiver operating characteristic (ROC) curves were used to determine the cutoff values for distinguishing between the two groups. Due to organ scarcity, it is recommended to accept all potentially viable kidneys [28, 29]. Therefore, cutoff values were defined to maximize specificity for identifying Group 2 kidneys. We adopted a significance level of 95%. We used Matlab 2023a (The MathWorks, Natick, MA, USA) and GraphPad Prism 9.3.1 (GraphPad Software Inc., San Diego, CA) for data processing, statistical analysis, and data presentation.

## 3 Results

All kidneys underwent 2 h of NMP without macroscopic abnormalities. Based on the pre‐set criteria, 7 kidneys were classified into Group 1 and 15 kidneys into Group 2. The average WITs were 35 min for Group 1 and 50 min for Group 2, and the CITs were 5.6 h and 6.4 h, respectively.

### 3.1 Renal Function and Viability Assessment

**FIGURE 2:**
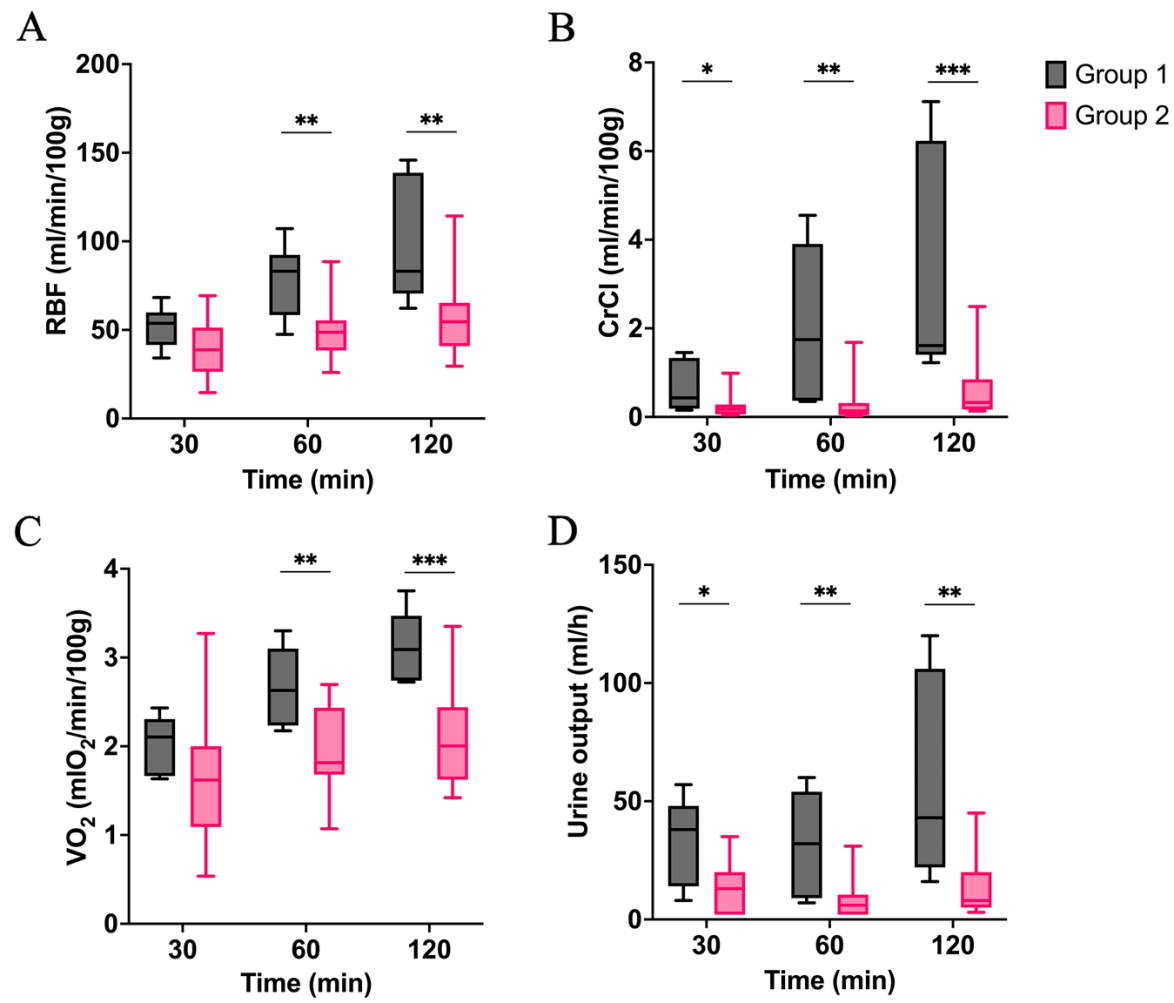
Function and viability parameters measured during NMP. (A) Renal blood flow (RBF); (B) Creatinine clearance (CrCl); (C) Oxygen consumption (VO_2_); (D) Urine output. *p ≤ 0.05, **p ≤ 0.01, ***p ≤ 0.001. [Color figure can be viewed at wileyonlinelibrary.com]

RBF demonstrated significant variations between the two groups. Specifically, at 30, 60, and 120 min, RBF was 54 (45–58), 83 (66–88), and 83 (76–128) ml/min/100 g in Group 1, and 39 (29–50), 49 (42–55), and 55 (43–65) ml/min/100 g in Group 2 (*p* = 0.078, *p* = 0.005, and *p* = 0.003, respectively, Figure 2A). CrCl showed a significant difference from 30 min [0.43 (0.20–1.25) vs. 0.17 (0.06–0.28) ml/min/100 g, *p* = 0.039] until 120 min [1.61 (1.46–4.27) vs. 0.33 (0.17–0.79) ml/min/100 g, *p* < 0.001, Figure 2B]. VO_2_ diverged significantly by 60 min of NMP, with Group 1 displaying a higher VO_2_ of 2.63 (2.34–3.03) mlO_2_/min/100 g compared to 1.82 (1.69–2.19) mlO_2_/min/100 g in Group 2 (*p* = 0.004, Figure 2C). Urine output was also higher in Group 1, particularly at 120 min [43 (28–85) vs. 8 (6–18) mL/h, *p* = 0.002, Figure 2D].

### 3.2 Power Doppler Metrics Assessment

**FIGURE 3:**
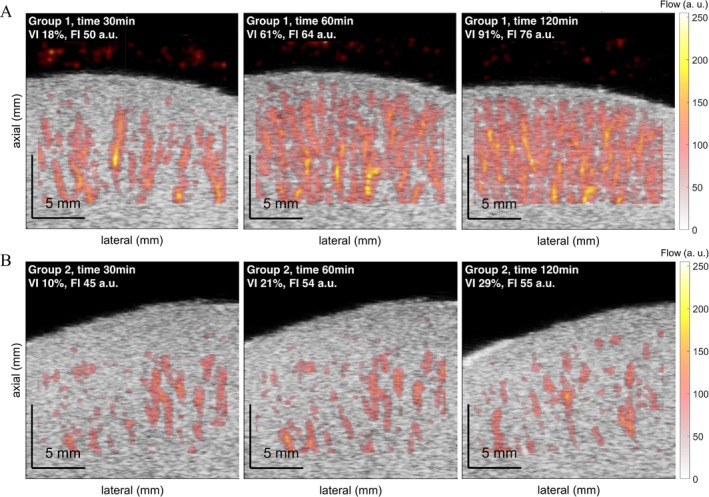
Representative Power Doppler (PD) imaging frames of renal cortex in (A) Group 1 and (B) Group 2 at 30, 60, and 120 min of NMP. FI, flow index; VI, vascularization index. [Color figure can be viewed at wileyonlinelibrary.com]

Representative PD imaging frames from both groups are shown in Figure 3. PD images distinctly discriminate the differences in renal cortical perfusion between the groups during NMP. In Group 1, PD images showed a progressive increase in signal intensity and vascularization over time, suggesting effective restoration of perfusion. In contrast, Group 2 demonstrated less improvement in perfusion recovery, as indicated by lower signal intensity and vascularization percentages.

**FIGURE 4:**
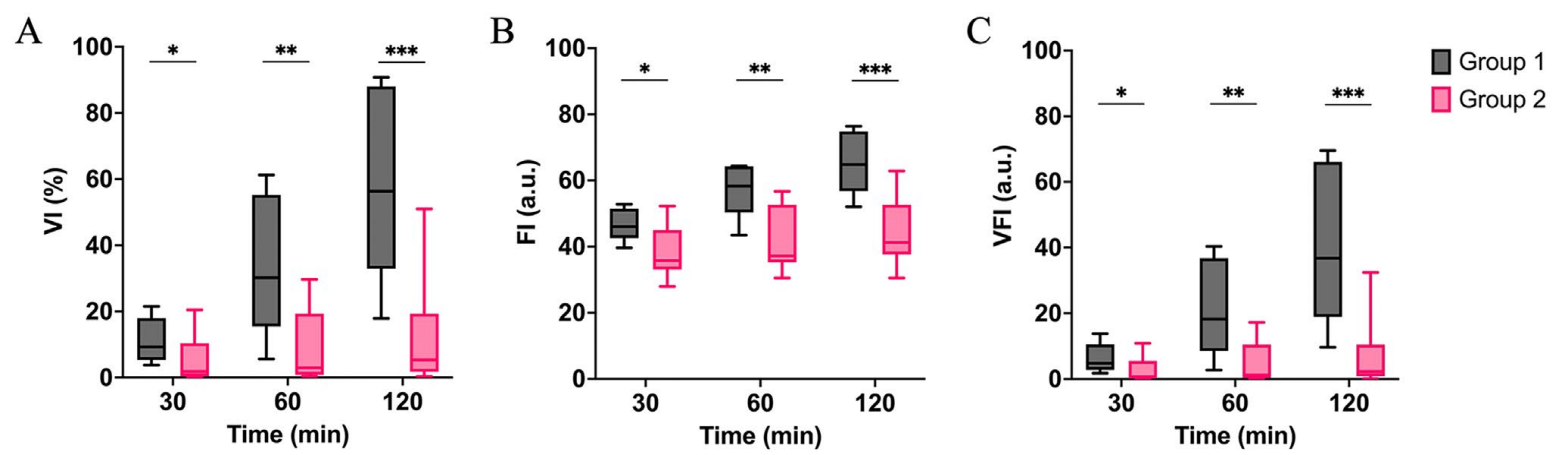
Quantitative PD metrics showing significant differences between Group 1 and Group 2 at 30 min of NMP. (A) Vascularization index (VI); (B) Flow index (FI); (C) Vascularization flow index (VFI). *p ≤ 0.05, **p ≤ 0.01, ***p ≤ 0.001. [Color figure can be viewed at wileyonlinelibrary.com]

The values of VI, FI, and VFI are depicted in Figure 4. VI differed significantly between the two groups at 30 min of NMP [9 (7–17)% vs. 2 (1–8)%, *p* = 0.026, Figure 4A]. FI also differed between the two groups. Group 1 showed a significantly higher FI at 30 min, indicating more robust perfusion compared to Group 2 [46 (44–51) vs. 36 (33–44) a.u., *p* = 0.032, Figure 4B]. VFI showed similar trends, with Group 1 exhibiting a significantly higher VFI at 30 min [5 (4–10) vs. 1 (0–4) a.u., *p* = 0.026, Figure 4C].

### 3.3 Correlation Between RBF and Functional Metrics

**FIGURE 5:**
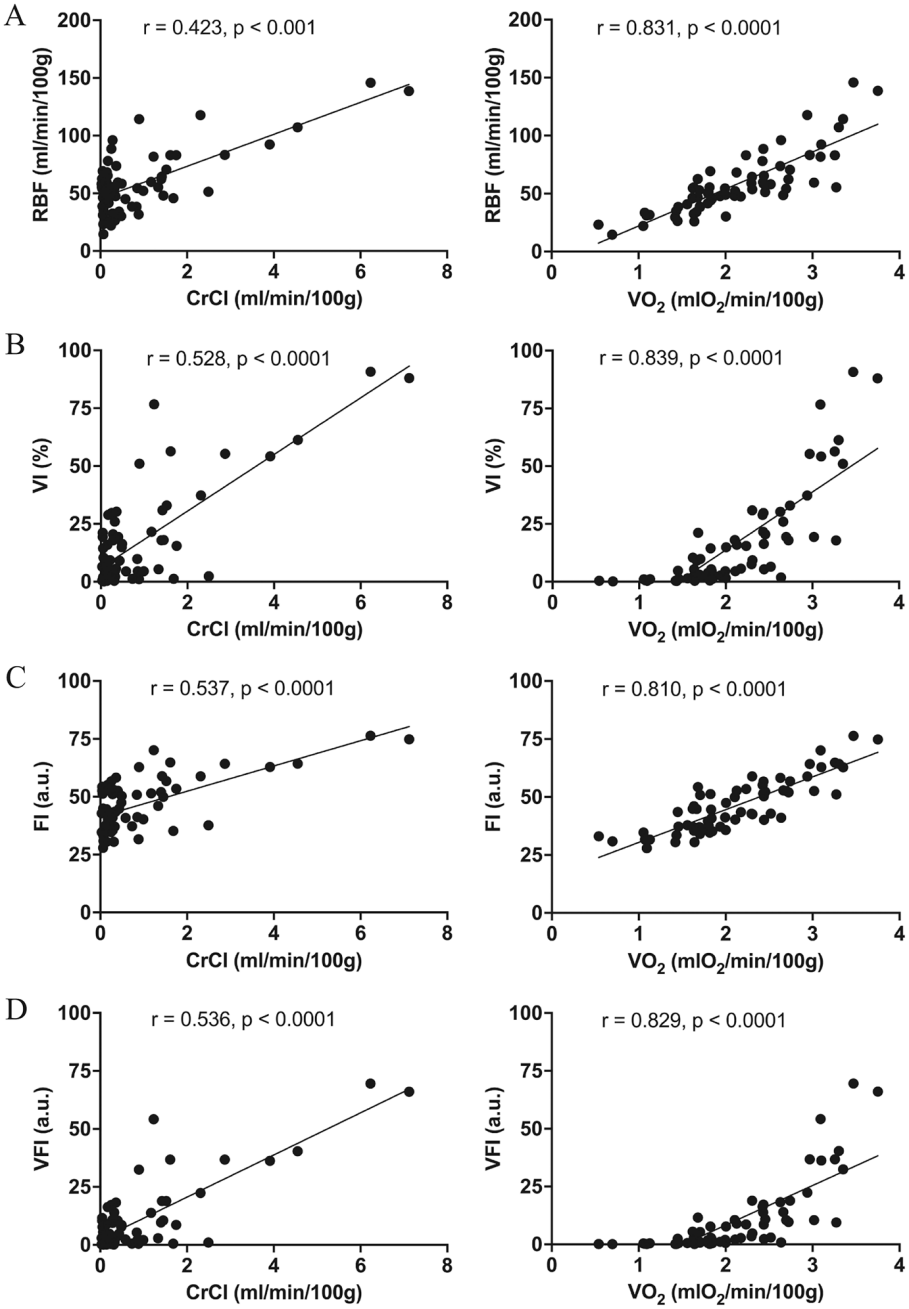
Correlation between (A) RBF, (B) VI, (C) FI, (D) VFI and CrCl (left) and VO_2_ (right). Black scatters represent all kidneys at all measured time points.

We investigated the correlation between renal perfusion velocities and functional parameters as depicted in Figure 5. Both RBF and PD metrics demonstrated significant positive correlations with CrCl and VO2. Specifically, while the correlation between RBF and CrCl was low positive (*r* = 0.423, *p* < 0.001), PD metrics demonstrated moderate correlations with CrCl (VI, *r* = 0.528, *p* < 0.0001; FI, *r* = 0.537, *p* < 0.0001; VFI, *r* = 0.536, *p* < 0.0001). Notably, both RBF and PD metrics showed strong positive correlations with VO_2_, while VI exhibited the highest correlation coefficient (*r* = 0.839, *p* < 0.0001).

### 3.4 Cutoff Values for Intergroup Differentiation

The ROC curve analyses of PD metrics and RBF at 30, 60, and 120 min of NMP are shown in Figures S1, S2, and Tables S3, S4. Among these time points, PD metrics at 120 min showed the most robust cutoff values for distinguishing between the two groups, with an area under the curve (AUC) of 0.943 for VI, 0.924 for FI, and 0.943 for VFI. Similarly, RBF at 120 min had the highest AUC of 0.886 (*p* = 0.004).

To minimize the risk of rejecting kidneys associated with favorable post‐transplant outcomes, we determined cutoff values that achieved 100% specificity for identifying Group 2 kidneys. A cutoff value of 60 mL/min/100 g for RBF shows 67% sensitivity, a negative predictive value (NPV) of 58%, and overall accuracy of 77%, while cutoff values of 17% for VI, 50 a.u. for FI, and 9 a.u. for VFI demonstrated 73% sensitivity, NPVs of 64%, and improved diagnostic accuracy of 82%.

### 3.5 Renal Injury Assessment

**FIGURE 6:**
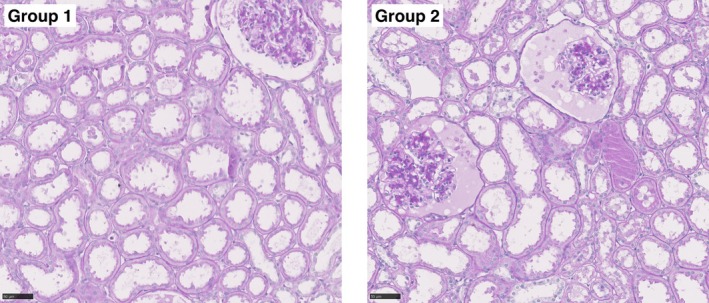
Representative periodic‐acid‐Schiff (PAS) staining of Group 1 (left) and Group 2 (right) prior to NMP. 40× magnification. [Color figure can be viewed at wileyonlinelibrary.com]

Representative histological images of kidney biopsies are depicted in Figure 6. In both groups, the tubules exhibited loss of brush border, debris, and lumen dilation, suggesting moderate ATN prior to NMP. No significant difference in the semiquantitative scale was observed between the groups.

## 4 Discussion

This study used a porcine kidney NMP model to investigate the value of PD imaging in assessing kidney quality. We observed significant differences in PD metrics between kidneys exhibiting high and low renal function during NMP. Our findings revealed a moderate positive correlation between PD metrics and renal function, and a strong positive correlation between PD metrics and renal viability. Compared to RBF, PD imaging may provide earlier detection of kidneys with poor posttransplant outcomes in this porcine model.

PD imaging has unique advantages in ease of use, real‐time monitoring, and relatively low cost, as it can be performed with standard ultrasound equipment without high operational costs and contrast agents. Additionally, PD imaging can be performed using a portable ultrasound system that costs under €1000. Since NMP is gradually being introduced into clinical practice in academic medical centers [6, 30], it is feasible to apply PD imaging for pre‐transplant kidney quality assessment in clinical settings.

In functional kidneys, PD imaging displays a diffused, homogeneous blush color across the entire renal cortex. In contrast, non‐functional kidneys significantly lower VI, FI, and VFI, indicating fewer blood‐filled vessels and reduced blood volume in the renal cortex. It can be explained by the prolonged WIT and high sensitivity to ischemic injury that results in high intrarenal resistance and inferior blood flow during machine perfusion [31, 32, 33].

Compared to RBF as routine monitoring during NMP, PD metrics differentiated kidneys in the two groups earlier. This discrepancy reflects the heterogeneity of the perfusion in the entire kidney (RBF) versus renal cortical perfusion status (PD), which is in line with the study by Schutter et al. [11] which reported heterogeneous corticomedullary ratios in kidneys with consistent RBF during NMP.

Post‐transplant PD imaging assessments can be affected by extrarenal variables rather than inherent renal injury. Renal cortical blood perfusion is easily affected by blood pressure, renal capillary wedge pressure, and vascular compliance, which reflect systemic hemodynamics [34]. In contrast, pre‐transplant assessment with standardized perfusion setups can reduce bias from extrarenal variables. We investigated the correlation between PD metrics and function and viability markers. VI, FI, and VFI showed a better correlation with CrCl than RBF. This can be explained by the fact that the majority of functional nephrons are located in the renal cortex. However, due to the complex mechanisms of kidney function, predicting renal function solely based on PD imaging is not ideal. These metrics need to be combined with other characteristics, biomarkers, and biophysical properties to accurately evaluate function. In terms of renal viability, both RBF and PD metrics showed a strong correlation with VO_2_, with VI displaying the highest correlation coefficient, indicating that PD metrics are reliable markers of renal metabolic activity.

Our results showed that, with appropriate cutoff values, both PD metrics and RBF can achieve 100% specificity in identifying kidneys with low renal function and viability during NMP, with PD showing higher overall accuracy. Based on this, kidneys classified as having low function and viability (Group 2) are recommended to be discarded. We also applied the ex vivo normothermic perfusion (EVNP) score, based on macroscopic appearance, RBF, and urine output on the kidneys. Group 1 kidneys scored 1 (*n* = 4) or 2 (*n* = 3), while Group 2 kidneys scored 2 (*n* = 8) or 3 (*n* = 7) (Table S5) [8]. Kidneys with an EVNP score of 2 present in both groups suggest an ambiguous threshold for transplant suitability. This overlap may also be attributed to the subjectivity of macroscopic assessment and inherent differences between porcine and human kidney perfusion.

We analyzed the correlation between PD metrics and EVNP scores. As shown in Figure S3, kidneys with EVNP scores 1, 2, and 3 exhibited distinct VI, FI, and VFI values, with the most significant differences observed at 120 min of NMP. At this timepoint, kidneys with an EVNP score of 1 demonstrated significantly higher PD metrics than those with an EVNP score of 3. This trend suggests that PD imaging may offer predictive value consistent with this clinically validated scoring system.

However, it is important to note that predictive accuracy based on PD metrics may vary depending on classification criteria, and relying solely on a single factor carries a risk of false‐negative predictions. Incorporating PD imaging into the current scoring system might enhance its accuracy and improve transplant decision‐making [8].

Interestingly, histological results revealed no significant differences between the groups. One explanation could be that the functional differences do not correlate with histological changes. In addition, due to sampling error: the local evaluation of the renal parenchyma may not represent the overall condition of the kidney [35].

The present study has several limitations to be addressed. First, while renal function and viability measured during NMP can reflect kidney quality, the absence of a reperfusion model with allogenic whole blood or a transplant model makes it impossible to determine the actual differences in post‐transplant kidney function between the two groups. Secondly, the use of slaughterhouse animals introduces great interindividual variability in kidney viability and injury due to the less controlled procedure, which could have influenced our outcome measurements. Thirdly, while porcine kidneys are commonly utilized in preclinical machine perfusion experiments, their anatomical and physiological characteristics differ from human kidneys. It is important to exercise caution when extrapolating our findings to clinical scenarios. Further research should focus on applying PD imaging to experimental kidneys intended for transplantation to validate our findings.

## 5 Conclusion

This study demonstrated the potential of PD imaging in pre‐transplant kidney quality assessment using a porcine kidney NMP model. The PD metrics strongly correlate with renal viability and can potentially identify kidneys with poor post‐transplant outcomes. PD imaging can be a valuable alternative to RBF during NMP. The explorative translation into clinical practice might help to optimize the utilization of marginal kidneys.

## Author Contributions

Yitian Fang participated in the research design, experiments, data analysis and interpretation, and drafting the manuscript. Anton V. Nikolaev participated in the research design, experiments, data analysis and interpretation, and reviewing the manuscript. Jeroen Essers, Gisela Ambagtsheer, and Marian C. Clahsen‐van Groningen participated in the experiments, data analysis and interpretation, and reviewing the manuscript. Robert C. Minnee, Ron W.F. de Bruin, and Gijs van Soest participated in the research design and critically reviewing the manuscript.

## Conflicts of Interest

Gijs van Soest was the PI on research projects conducted at Erasmus MC that received research support from FUJIFILM VisualSonics, Shenzhen Vivolight, Boston Scientific, Waters, and Mindray.

## Supporting information


Data S1.
