# Supplementary material for: Enhancing Kidney Quality Assessment: Power Doppler During Normothermic Machine Perfusion
Source: Artif Organs. 2025 Mar 12;49(7):1132–40. doi: 10.1111/aor.14983 (PMC12179758; doi:10.1111/aor.14983)
Supplement: Supplementary file 1 — Data S1. [file AOR-49-1132-s001.docx]

**Supplementary**

**Table S1. Components of the normothermic machine perfusion perfusate**

| **Priming** | **Volume (mL)** |
| --- | --- |
| Ringer’s lactate (Baxter, the Netherlands) | 300 |
| Autologous leukocyte-depleted blood | 500 |
| **Additives** |  |
| 8.4% sodium bicarbonate (B. Braun, Germany) | 10 |
| 5% glucose (Baxter, the Netherlands) | 10 |
| Verapamil (Sigma-Aldrich, the Netherlands) | 1 |
| Dexamethasone (Centrafarm, the Netherlands) | 0.3 |
| 90mg creatinine (Sigma-Aldrich, the Netherlands) | - |
| **Infusion 20mL/h** |  |
| Aminoplasmal (B. Braun, Germany) | 45 |
| 8.4% sodium bicarbonate (B. Braun, Germany) | 1.5 |
| 100 U/mL insulin (NovoRapid^®^, Denmark) | 0.5 |

**Table S2. Settings used for PD data acquisition**

| PRF (kHz) | Tx frequency (MHz) | Gate | Wall filter | Sensitivity | Dynamic range (dB) |
| --- | --- | --- | --- | --- | --- |
| 2 | 12.5 | 4 | High | 5 | 30 |

**Table S3. ROC curve analysis of PD metrics**

| Time | PD metrics | AUC | 95% CI | p value |
| --- | --- | --- | --- | --- |
| 30 min | VI (%) | 0.800 | 0.616-0.984 | 0.026 |
|  | FI (a.u.) | 0.791 | 0.603-0.978 | 0.032 |
|  | VFI (a.u.) | 0.800 | 0.616-0.984 | 0.026 |
| 60 min | VI (%) | 0.867 | 0.710-1.000 | 0.007 |
|  | FI (a.u.) | 0.886 | 0.742-1.000 | 0.004 |
|  | VFI (a.u.) | 0.867 | 0.710-1.000 | 0.007 |
| 120 min | VI (%) | 0.943 | 0.848-1.000 | 0.001 |
|  | FI (a.u.) | 0.924 | 0.814-1.000 | 0.002 |
|  | VFI (a.u.) | 0.943 | 0.848-1.000 | 0.001 |

AUC, area under the curve; 95% CI, 95% confidence interval; FI, flow index; PD, Power Doppler; VFI, vascularization flow index; VI, vascularization index.

**Table S4. ROC curve analysis of RBF**

| Time | AUC | 95% CI | p value |
| --- | --- | --- | --- |
| 30 min | 0.748 | 0.535-0.960 | 0.067 |
| 60 min | 0.867 | 0.692-1.000 | 0.007 |
| 120 min | 0.886 | 0.747-1.000 | 0.004 |

AUC, area under the curve; 95% CI, 95% confidence interval; RBF, renal blood flow.

**Table S5. Ex vivo normothermic perfusion assessment score of the kidneys**

| Group | Kidney | Macroscopic assessment^a^ | Renal blood flow (ml/min/100g)^b^ | Total urine output (ml)^c^ | EVNP score^d^ |
| --- | --- | --- | --- | --- | --- |
| Group 1 | K3 | Grade I | 74 | 15 | 2 |
| Group 1 | K4 | Grade I | 83 | 70 | 1 |
| Group 1 | K7 | Grade I | 58 | 23 | 2 |
| Group 1 | K13 | Grade I | 83 | 111 | 1 |
| Group 1 | K19 | Grade I | 107 | 108 | 1 |
| Group 1 | K20 | Grade I | 92 | 97 | 1 |
| Group 1 | K21 | Grade I | 50 | 26 | 2 |
| Group 2 | K1 | Grade I | 54 | 15 | 2 |
| Group 2 | K2 | Grade I | 49 | 26 | 3 |
| Group 2 | K5 | Grade I | 89 | 8 | 2 |
| Group 2 | K6 | Grade I | 63 | 7 | 2 |
| Group 2 | K8 | Grade I | 58 | 29 | 2 |
| Group 2 | K9 | Grade I | 46 | 39 | 3 |
| Group 2 | K10 | Grade I | 37 | 30 | 3 |
| Group 2 | K11 | Grade I | 50 | 4 | 2 |
| Group 2 | K12 | Grade I | 34 | 5 | 3 |
| Group 2 | K14 | Grade I | 45 | 66 | 2 |
| Group 2 | K15 | Grade I | 48 | 7 | 3 |
| Group 2 | K16 | Grade I | 26 | 18 | 3 |
| Group 2 | K17 | Grade I | 53 | 21 | 2 |
| Group 2 | K18 | Grade I | 55 | 11 | 2 |
| Group 2 | K22 | Grade I | 38 | 35 | 3 |

a. Grade I: excellent perfusion (global pink appearance, score 1); grade II: moderate perfusion (patchy appearance, score 2); grade III: poor perfusion (global mottled and purple/black appearance, score 3).

b. Threshold ≥ 50: score 0; threshold < 50: score 1.

c. Threshold ≥ 43: score 0; threshold < 43: score 1.

d. Ex vivo normothermic perfusion (EVKP) score is the sum of macroscopic assessment, renal blood flow and total urine output scores.

#
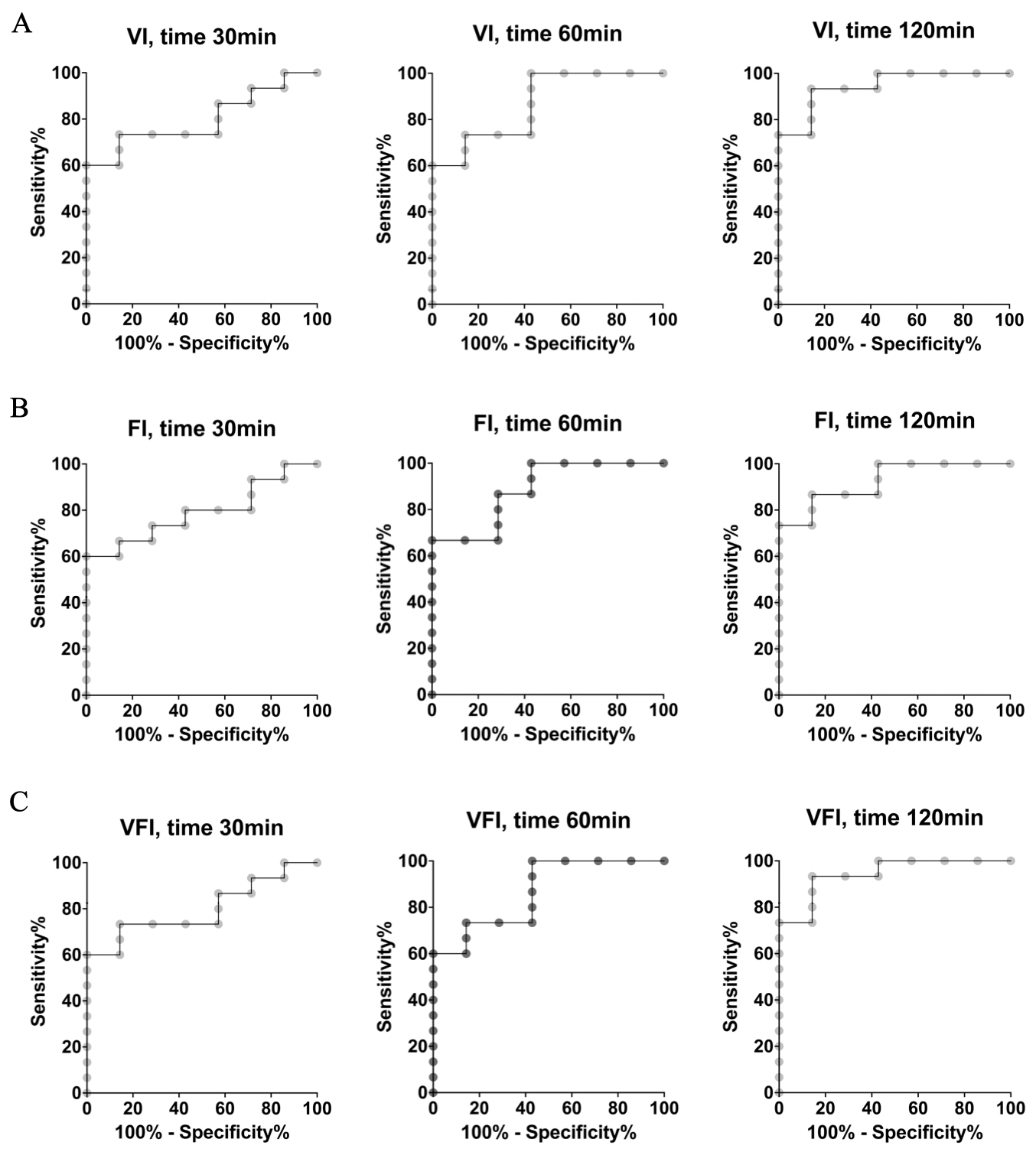


**Figure S1. Receiver operating characteristic (ROC) curves of (A) vascularization index (VI), (B) flow index (FI), and (C) vascularization flow index (VFI) to differentiate between the functional and non-functional kidneys at 30, 60, 120 minutes of NMP.**


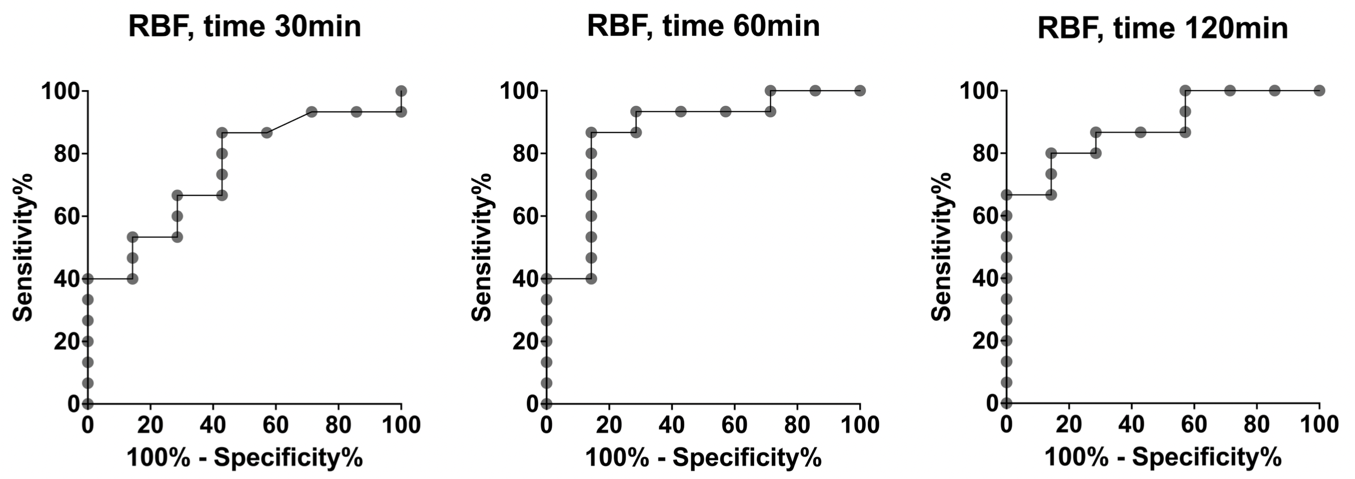


**Figure S2. Receiver operating characteristic (ROC) curves of renal blood flow (RBF) to differentiate between the functional and non-functional kidneys at 30, 60 and 120 minutes of NMP.**

**
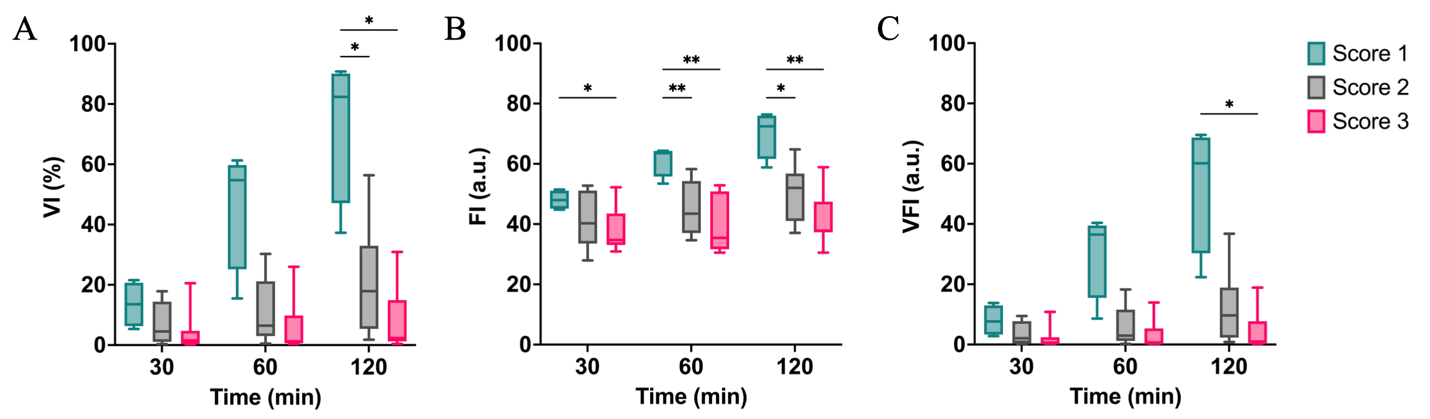
**

**Figure S3. Power Doppler metrics of the kidneys classified by ex vivo normothermic perfusion (EVNP) score during NMP.** *p≤0.05, **p≤0.01, ***p≤0.001.
